
JOURNAL INFORMATION
==============================
NLM Title Abbreviation: ScientificWorldJournal
Journal ID: ScientificWorldJournal
NLM Journal ID: 101131163
Journal ID: TSWJ

The Scientific World Journal

ISSN: 2356-6140
EISSN: 1537-744X
Publisher: Wiley

ARTICLE INFORMATION
==============================
PMCID: PMC6084250
PMID: 30147504
DOI: 10.1100/tsw.2001.167
Article ID: 584938
Article version: 1
Subjects: Short Report

B709 Mitochondrial Control of Cell Death

Kroemer Guido
CNRS-UMR1599, Institut Gustave Roussy, F-94805 Villejuif, France
Publication date: 2001
Electronic publication date: 2001 Oct 5
Volume: 1
First page: 48
Last page: 48
Copyright: Copyright © 2001 Guido Kroemer.
Copyright year: 2001
License: This is an open access article distributed under the Creative Commons Attribution License, which permits unrestricted use, distribution, and reproduction in any medium, provided the original work is properly cited.
License URL: https://creativecommons.org/licenses/by/3.0/

==============================
Miami *Nature Biotechnology* Short Reports
*TheScientificWorld* (2001) 1(S3), 48SR
ISSN 1532-2246; DOI 10.1100/tsw.2001.167

### B709 MITOCHONDRIAL CONTROL OF CELL DEATH

Guido Kroemer

CNRS-UMR1599, Institut Gustave Roussy, F-94805 Villejuif, France
kroemer@igr.fr

**INTRODUCTION.** Mitochondrial membrane permeabilization (MMP) is a critical event of early apoptosis. MMP is regulated by pro- and anti-apoptotic members of the Bax/Bcl-2 family of proteins, via a process which may involve sessile mitochondrial proteins organized in the two membrane-spanning permeability transition pore complex (PTPC). Apoptotic MMP differentially affects the outer and the inner mitochondrial membranes (1-3). The inner mitochondrial membrane becomes permeable to solutes up to 1500 Da, yet retains matrix proteins in their normal localization. In contrast, the outer mitochondrial membrane becomes completely permeabilized to proteins, leading to the release of soluble proteins from the mitochondrial intermembrane space to an ectopic (extra mitochondrial) localization. Several intermembrane proteins can activate catabolic hydrolases involved in the apoptotic process (4). One such protein is cytochrome *c*, which participates in the caspase activation cascade. Another functionally important intermembrane protein is apoptosis inducing factor (AIF), which functions as a caspase-independent death effector (5).

**AIF EXPRESSION AND LOCALIZATION.** AIF is strongly conserved among mammalian species (92% aa identity in the whole protein between mouse and man). The mouse AIF cDNA codes for a protein which is organized in three domains (i) an amino-terminal mitochondrial localization sequence (MLS) of 100amino acids; (ii) a spacer sequence of 27aminoacids; and (iii) a carboxyterminal 485 amino acid domain with strong homology to oxido reductases from other vertebrates (*X. laevis*), non-vertebrate animals (*C. elegans*, *D. melanogaster*), plants, fungi, eubacteria, and archaebacteria (6). The AIF gene is located on the X chromosome (Xq24/225) and is ubiquitously expressed, both in normal tissues and in a variety of cancer cell lines (7). The mature AIF protein, a flavoprotein (prosthetic group: FAD) with significant homology to plant ascorbate reductases and bacterial NADH oxidases, is normally confined to the mitochondrial intermembrane space (5,8). Accordingly, transient transfection of COS cells with green fluorescent protein (GFP) fused to the C-terminus of AIF to generate a chimeric AIF-GFP protein, targets GFP to mitochondria (9). When cells are induced to undergo apoptosis, AIF is released from mitochondria to the cytosol and translocates to the nucleus. This has been confirmed using all available methods for the determination of the subcellular localization of AIF: in situ immunostaining (5,8,10), transfection with AIF-GFP (9), and subcellular fractionation (5,10).

**AIF AS A NOVEL DEATH EFFECTOR.** When added to purified nuclei, AIF induces peripheral nuclear chromatin condensation, as well as large scale (~50 kbp) DNA fragmentation. If microinjected into the cytoplasm of normal cells, recombinant AIF suffices to cause several hallmarks of apoptosis including the condensation of nuclear chromatin and the exposure of phosphatidylserine on the plasma membrane surface (5,9,10). These alterations are rapid (30-120 min) and are not prevented by addition of the pan-caspase inhibitor Z-VAD. Fmk (5,10). Moreover, they are not affected by over expression of the anti-apoptotic protein Bcl-2 (5). Similar in vivo effects have been obtained by transfection-enforced over expression of a truncated AIF-GFP construct misdirected to the extra-mitochondrial compartment due to the deletion of the N-terminal MLS (AIF-GFPD1-100) (9). Thus, extra mitochondrially targeted AIF is a dominant death inducer.

**CONTRIBUTION OF AIF TO CELL DEATH.** The intracellular neutralization of extra mitochondrial AIF by microinjection of an AIF-specific antibody has established two alternative models of the AIF involvement in cell death. In mouse embryonic fibroblasts, AIF neutralization only abolishes nuclear apoptosis when theapoptosome/caspase-3/CAD pathway is simultaneously blocked by genetic invalidation of Apaf-1 or caspase-3, addition of the pan-caspase inhibitor Z-VAD.fmk, or microinjection of ICAD (11). Such data indicate the co-existence of two alternative pathways linking mitochondria to nuclear apoptosis, one that requires AIF and another that relies on caspase activation. In sharp contrast, in other models of cell death (Rat-1 cells treated with staurosporin or HeLa cells dying upon interaction between the HIV-1envelope glycoprotein complex and CD4/CXCR4), microinjection of AIF antibody blocks the release of cytochrome *c* and subsequent caspase activation (while cytochrome *c* neutralization and caspase inhibition do not affect the release of AIF), placing AIF upstream of the cytochrome *c*/Apaf-1/caspase/CAD pathway (5,10). Accordingly, when added to purified mitochondria in combination with cytosolic extract, recombinant AIF causes the permeabilization of the outer mitochondrial membrane, leading to the release of cytochrome *c* and subsequent caspase activation (5). Moreover, transfection-enforced overexpression of AIF in Rat-1 cells (9) or microinjection of recombinant AIF into HeLa syncytia (10) can trigger the release of cytochrome *c* from mitochondria. These results have been confirmed in embryonic stem cells in which the AIF gene has been ablated.

**CONCLUSION.** AIF may act as a facultative signaling molecular upstream (or at the level) of mitochondria, as well as death effector downstream of mitochondria. More over, our data underline that mitochondria contribute to apoptosis regulation via a variety of mechanisms.

**ACKNOWLEDGEMENT.** This work is supported by a special grant from the Ligue Nationale contre le Cancer, as well as by grants from ANRS, FRM, IGR, and EC.

**REFERENCES.**

1. Kroemer, G. and Reed, J.C. (2000) Nat. Med. 6, 513-519
2. Costantini, P., Jacotot, E., Decaudin, D., and Kroemer, G. (2000) J. Natl. Cancer Inst. 92, 1042-1053
3. Zamzami, N. and Kroemer, G. (2001) Nat. Rev. Mol. Cell. Biol. (in press)
4. Patterson, S. et al. (2000) Cell Death Differ. 7, 137-144
5. Susin, S.A. et al. (1999) Nature 397, 441-446
6. Lorenzo, H.K., Susin, S.A., Penninger, J., and Kroemer, G. (1999) Cell Death Differ. 6, 516-524
7. Daugas, E. et al. (2000) FEBS Lett. 476, 118-123
8. Daugas, E. et al. (2000) FASEB J. 14, 729-739
9. Loeffler, M. et al. (2001) FASEB J. (in press)
10. Ferri, K.F. et al. (2000) J. Exp. Med. 1, 1081-1092
11. Susin, S.A. et al. (2000). J. Exp. Med. 192, 577-585
